# Experimental evaluation of cryopreservative solutions to maintain *in vitro* and *in vivo* infectivity of *P*. *berghei* sporozoites

**DOI:** 10.1371/journal.pone.0177304

**Published:** 2017-05-22

**Authors:** Naresh Singh, Samantha J. Barnes, Sandra Kennedy, John H. Adams

**Affiliations:** Department of Global Health, College of Public Health, University of South Florida, Tampa, Florida, United States of America; INSERM, FRANCE

## Abstract

The rodent malaria parasite *Plasmodium berghei* is an excellent model organism for laboratory-based experimental evaluation of anti-malarial therapeutics prior to studies with human malaria parasites. The rodent model is especially important for evaluation of pre-erythrocytic (PE) stage therapies, especially as current efforts to develop new PE vaccines and drugs is limited by access to *P*. *falciparum* and *P*. *vivax* sporozoites. Developing a more effective method for cryopreservation of sporozoites would help improve access to sporozoites for laboratories lacking suitable insectary facilities. In this study, *P*. *berghei* GFP-expressing sporozoites were purified from infected mosquitoes by manual dissection of salivary glands and different commercially-available, serum-free cryopreservative solutions were evaluated for efficient cryopreservation of the sporozoites. The cryopreservative solutions evaluated included CryoStor CS2, CryoSolutions DX5, CryoSolutions MC, Hestar 200, Voluven, Hetastarch, and Glycerolyte 57. The viability of fresh and post-thaw cryopreserved sporozoites was determined as a function of the relative sporozoite infectivity by infecting HC-04 cells *in vitro*, monitoring invasion and growth and development of liver stage parasites. Flow cytometer-based counting provided unbiased and fast quantitative assessment of parasite *in vitro* infection in infected HC-04 and *in vivo* infectivity was validated by injecting sporozoites IV into mice. CryoStor CS2 delivered the highest post-thaw recovery and infectivity of cryopreserved sporozoites. Sporozoites cryopreserved in CryoStor CS2 achieved 38% complete development of hepatic stages in HC-04 and 100% infectivity in mice. The cryopreservation method described here demonstrates a viable alternative for fresh *Plasmodium* sporozoites. The use of cryopreserved sporozoites should facilitate greater access to sporozoites for chemotherapeutic and vaccine research.

## Introduction

Malaria remains one of the deadliest human diseases affecting millions of people worldwide, especially in many tropical countries where mosquito vectors are prevalent and access to health care is more limited [1]. The major causes of malaria in most endemic countries are *Plasmodium falciparum* in Africa and *P*. *vivax* outside of Africa. Mosquitoes feeding on a person with malaria become infected with *Plasmodium* sexual stages and subsequently complete transmission about 2 weeks later by biting another person. Sporozoites travel through blood circulation to reach the liver and infect hepatocytes to initiate an exoerythrocytic cycle for a clinically silent hepatic stage. In the liver sporozoites transform by asexual multiplication process to produce thousands of merozoites [2] and initiate the disease-causing blood-stage cycle.

Targeting therapies to block mosquito transmission is critical for control and elimination of malaria, but this phase is difficult for laboratory research due to the need of specialized facilities and the need for highly skilled laboratory staff. Distribution of infected mosquitoes is also limited due to biosafety concerns and since sporozoites, once inside salivary glands, are only infective to human host for a short time [3]. Consequently, research on sporozoites and liver stages of human malaria parasites is limited for experimental studies. Alternatives to research with the mosquito stages of human malaria parasites include several rodent malaria parasites (*P*. *berghei*, *P*. *chabaudi*, *P*. *vinckei*, *P*. *yoelii*) that are in widespread use. These models are relatively easy to work with and they display substantial genotype and phenotype similarities to the human malaria parasites *P*. *falciparum* and *P*. *vivax* [4]. Rodent malaria models have long been utilized as model for human malaria species, although they also can require substantive investments in infrastructure. In this study, we explore cryopreservation of sporozoites as a potential alternative to the limited availability of fresh sporozoites.

Commercially-available cryopreservative solutions usually contain at least one active ingredient, such as DMSO, glycerol, and/or hydroxyethyl starch. These components help maintain cell integrity and viability by limiting ice crystal formation during freezing and post-thaw recovery. In addition, cryopreservative solutions contain chemicals that maintain buffering capacity to minimize extreme pH cellular damage. Relatively few studies have explored methods to improve cryopreservation methods for *Plasmodium* sporozoites. Early studies that attempted to cryopreserve sporozoites of the human malaria parasite *P*. *falciparum* reported mixing sporozoites with plasma at -70C for several days [5]. While others reported cryopreservation of *P*. *vivax* sporozoites premixed with 50% FBS in saline stored in liquid N2 without any cryoprotectant and tested viability in monkey model [6]. Weathersby and McCall (1967) reported preservation of *P*. *gallinaceum* sporozoites in liquid nitrogen and evaluated in chickens [7]. Studies that cryopreserved *P*. *berghei* sporozoites used DMEM supplemented with 10% hydroxyethyle starch and 50% mouse serum but with very low success of post-thaw viability (6.8% for fresh and 0.5% for cryopreserved post-thaw) [8]. Alternatively, *P*. *berghei* sporozoites were cryopreserved using only mouse serum or a combination of medium with serum and DMSO or starch, suggesting various methods can be used successfully for sporozoite cryopreservation, although positive results were only determined by *in vivo* infectivity for mice [9].

Previously, we used the cryopreservative solutions listed to evaluate their capacity to successfully cryopreserve *P*. *falciparum* and *P*. *vivax* sporozoites, relying on the standard *in vitro* gliding assay as a measure of viability [10]. The study presented here extend these cryopreservation studies, using *P*. *berghei* GFP expressing sporozoites [11], to evaluate efficacy for maintaining *in vitro* and *in vivo* hepatocyte infectivity. The efficiency of cryopreservation and viability of sporozoites evaluated by infecting HC-04 [12] hepatocytes *in vitro* using flow cytometer based quantitative determination method and in mice with fresh and thawed cryopreserved sporozoites. The method successfully provides a simple and viable approach that minimize loss of viability with maximum recovery of infective sporozoites.

## Materials and methods

### Production of *P*. *berghei* ANKA GFP infected mosquitoes

*Plasmodium berghei* ANKA GFP (MRA888, MR4) line was maintained in ICR mice [11]. Mice were monitored for blood stage infection and development of gametocytes in Giemsa-stained blood smears. *Anopheles stephensi* (day 4–6 old) mosquitoes were fed on mice when parasitemia was 5–10%. Blood-fed infected mosquitoes were maintained on 10% sugar solution at 21C and 75–80% humidity in an environmental chamber set to a 12/12 hr light/dark cycle. Mosquito infectivity was determined by counting midgut oocyst after 8 days post blood meal. *An*. *stephensi* colony mosquitoes were bred in lab according to MR4 methods [13].

### Mosquito dissection and sporozoites cryopreservation

The procedures for mosquito dissection and purification of sporozoites is similar to that published recently [10]. Briefly, infected mosquitoes were chilled at -20C for 5 min and washed with 70% ethanol followed by rinse in 1xPBS containing mixture of penicillin (50 IU), streptomycin (50μg/ml) and neomycin (0.025 μg/ml) (GIBCO) before dissection. Mosquito salivary glands were removed by manual dissection and collected in sterile incomplete RPMI1640 (Corning) on day 19–21 post blood meal. Sporozoites were isolated by trituration of pooled salivary glands in each batch with plastic pestle in a microcentrifuge tube in 100μl incomplete RPMI1640. Sporozoites counts were made by hemocytometer to determine yield. Sporozoites were maintained on ice after isolation. Sporozoites suspension was prepared in incomplete RPMI1640 at 10,000 sporozoites/μl and mixed with chilled cryopreservative solutions (Table 1). The mixture of sporozoites were dispensed in 100μl aliquots with 250,000 sporozoites per cryogenic vial (Matrix). Samples were incubated on ice for 30 min followed by incubation at -80C for one hour. Cooled and frozen sporozoites samples were immediately transferred to liquid nitrogen vapor phase (LNVP) for long-term storage (Cryoplus 3, Thermo Fisher).

**Table 1:** Product information and important applications about cryogenic solutions.

Cryogenic Solutions	Vendors	Catalog Numbers	Important Component(s)	Major Applications
CryoStor CS2	Sigma	C3124	2% DMSO	Cryopreservation of stem cells, hepatocytes
CryoSolutions DX5	AKRON	AK9544	5% DMSO, 5% Dextran 40	Cryopreservation of stem cells, mammalian cells, biological tissues
CryoSolutions MC	AKRON	AK8995	10% DMSO, Methylcellulose	Cryopreservation of stem cells, mammalian cells, biological tissues
Hestar 200	Claris	G/LVP-5	6% Hydroxyethyl starch (200/0.5)	Blood plasma substitute
Voluven	Hospira	PB-1909	6% Hydroxyethyl starch (130/0.4)	Blood plasma substitute
Hetastarch	B Braun	L6511	6% Hydroxyethyl starch (450/0.75)	Blood plasma substitute
Glycerolyte 57	Fenwal	4A7833	50–60% Glycerin	Cryopreservation of blood stage Plasmodium

### Maintenance of HC-04 cells and infection with *Plasmodium berghei* GFP sporozoites

Hepatocytoma HC-04 cells (MRA-975) [12] were grown in collagen coated small culture flask in F12 and MEM (Invitrogen) medium supplemented with 10% FBS (Hyclone) and 1x pen-strep-neo (GIBCO) incubated at 5% CO_2_ and 37C. For experimental use cells were seeded (80,000/well) one day prior to infection with sporozoites in 96 well plate. Purified sporozoites were diluted in complete HC-04 medium to infect HC-04 cells in 50μl/well volume (40,000 sporozoites/well). Each infection was performed with 4 replicates and data were collected from two independent experiments for each test condition. After addition of sporozoites to the cells culture plate was centrifuged at 1931 X g, 5 min to ensure uniform contact of sporozoites to the cells [14]. Cell culture plate were incubated at 5% CO_2_ and 37C for 3 hrs. After 3 hrs un-invaded free sporozoites were washed 3 times with HC-04 medium or 1xPBS to prevent further invasion. Infected HC-04 cells were stained with DRAQ5 (Fisher) diluted (1:500) in trypsin EDTA (GIBCO) for 10 min at 37C while attached in wells. Cells were harvested in HC-04 medium and counted on Accuri flow cytometer version 1.32 (BD Biosciences) and analyzed by CFlow plus software. It is equipped with 50 mW 488 nm blue laser and 30 mW 640 red laser with excitation/emission spectra 488nm/640nm. Stained cells were gated in FL-1A (GFP, 488 nm) and in FL-4A (DRAQ5, 640 nm).

### Determination of infectivity and viability of fresh and post-thaw cryopreserved sporozoites in HC-04 cells

Sporozoites were used immediately (“fresh”) to infect HC-04 cells to establish the (control) baseline infectivity and also cryopreserved as described above. Cryopreserved sporozoites samples were revived by two different thawing methods. Samples were removed from LN storage and (method 1) held at room temperature or (method 2) dipping vial in 37C water bath until ice crystals disappeared. Next, samples were diluted immediately with an equal volume of complete hepatocyte medium and counted in a hemocytometer to calculate the number of recovered sporozoites (by formula below).

$$\text{Sporozoites recovery }\left(\text{\%}\right)=\;\frac{\text{Number of sporozoites counted post}-\text{thaw}}{\text{Number of sporozoites before cryopreservation}}\text{x}100$$

The number of cryopreserved sporozoites was adjusted by addition of hepatocyte medium and HC-04 cells were infected with 40,000 sporozoites/well in 4 replicates. Sporozoite infectivity was calculated as the percentage of infected HC-04 cells (by formulas below).

$$\text{Infected HC}-04\;\left(\text{\%}\right)=\frac{\text{Sporozoites infected HC}-04\text{ cells only}}{\text{Total counted cells}}\text{x}100$$

Relative infectivity of cryopreserved sporozoites infecting HC-04 cells was used as a primary determinant of viability (by formula below).

$$\text{Relative sporozoite infectivity }\left(\text{\%}\right)=\;\frac{\begin{array}{c} \text{Percent infected HC}-04\text{ of cryopreserved sporozoites post}-\text{thaw} \end{array}}{\text{Percent infected HC}-04\text{ of fresh sporozoites}}\times 100$$

Also, cryopreserved sporozoites were evaluated by invasion, growth and development in extended period of incubation and samples were collected at 3 hrs, day 1, day 2 and day 3 post infection and counted by flow cytometer. The growth and development rate over time to produce mature schizonts was defined as progression rate (by formula below).

$$\text{Progression rate }\left(\text{\%}\right)=\;\frac{\text{Viability on next day}}{\text{Viability on previuos day}}\text{ x }100$$

### *In vivo* infectivity of *P*. *berghei* sporozoites

Mice were injected with fresh or thawed cryopreserved sporozoites. Sporozoites in complete hepatocyte medium, as prepared for *in vitro* assays, were injected 100μl/mice intravenously into a tail vein. In each group 2 mice per dose were injected and data were collected from two independent experiments. Mice were followed for blood stage infection post inoculation by Giemsa-stained thin blood smears. Infected mice were observed for pre-patent period with the appearance of first blood stage parasites. All animals were euthanized by exposing to increasing dose of CO2 until complete cessation of respiratory function and confirmed death. All animals used in the study were approved by USF Institutional Animal Care and Use Committee (IACUC) protocol (R0242).

### Statistical analysis

All data were tested for normality with the Anderson-Darling test prior to analysis. The statistical difference between groups was determined using the 1-way analysis of variance for data following a normal distribution and the Kruskal-Wallis for data not following a normal distribution. A Bonferroni multiple comparison test was used to identify the statistical differences between groups. All statistical analysis was performed using SAS software (Cary, NC).

## Results

### Standardization of conditions to evaluate cryopreservation protocols using *P*. *berghei* sporozoites

**Fig 1:**
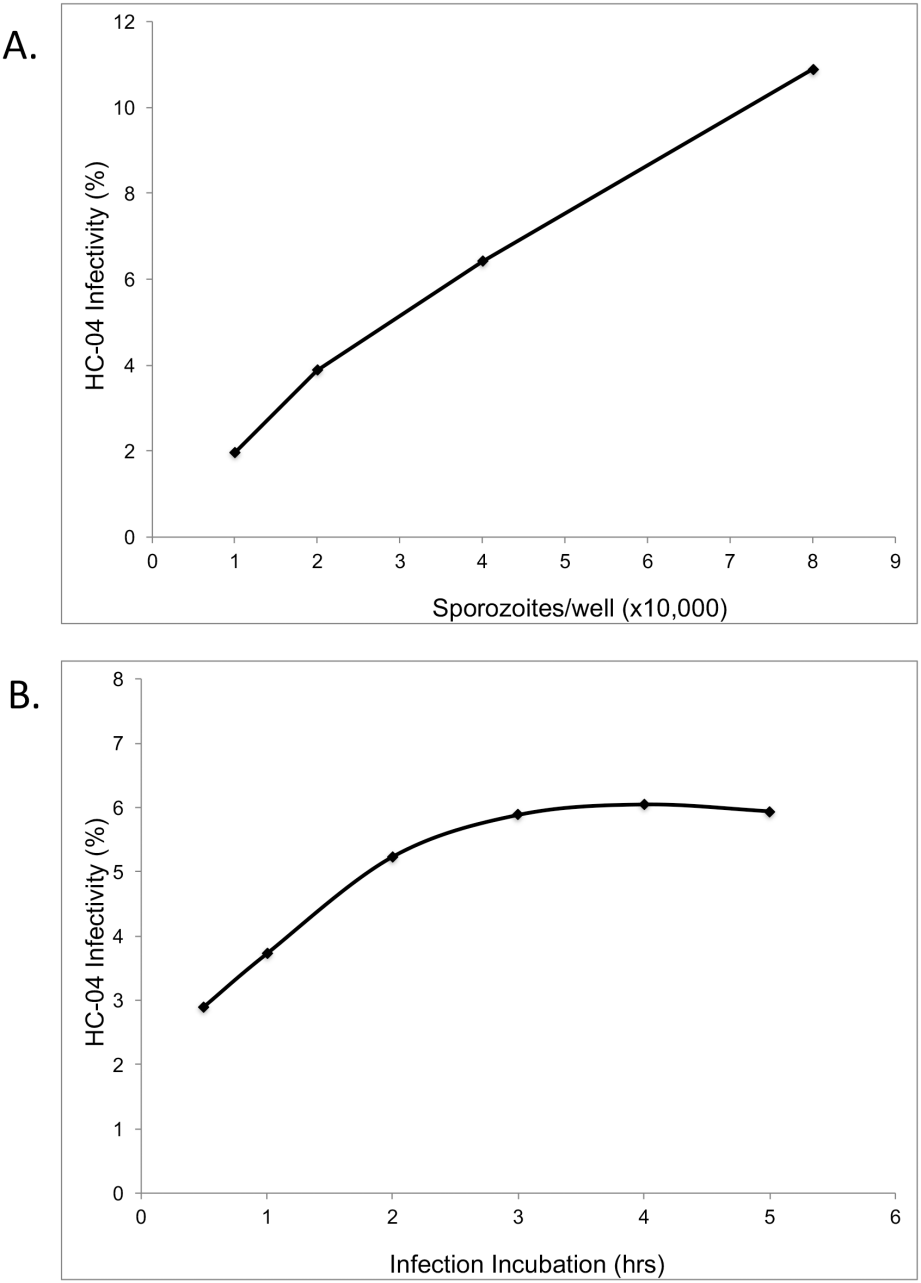
Infectivity of fresh *P*. *berghei* GFP sporozoites to HC-04 cells. (A) The infectivity of HC-04 cells with fresh sporozoites is shown for increasing numbers of sporozoites, using 80,000 HC-04 cells per well. (B) The infectivity of HC-04 cells with fresh sporozoites is shown while varying the infection incubation time, using 40,000 sporozoites and 80,000 HC-04 cells per well.

**Fig 2:**
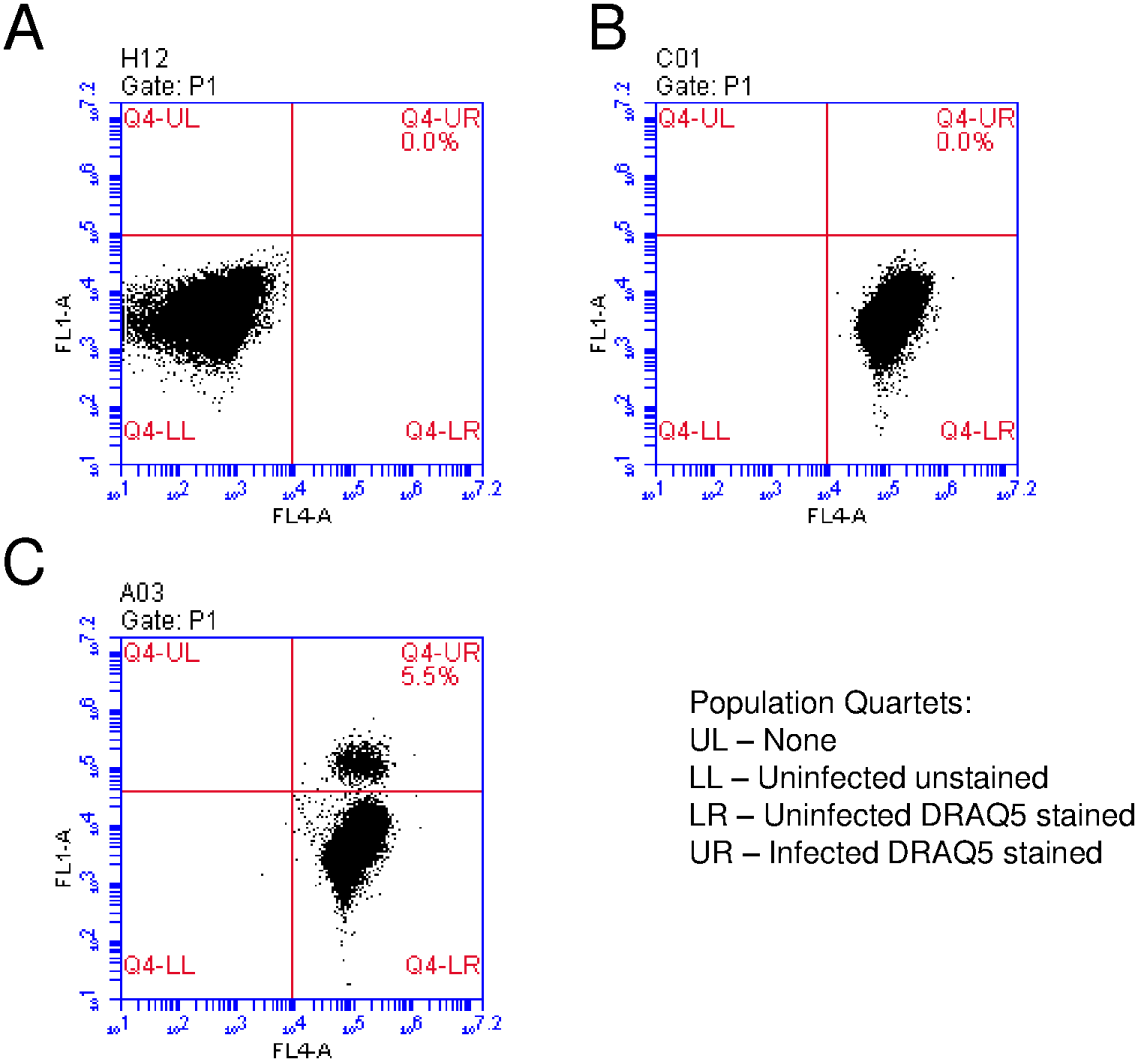
Optimization of flow cytometer program. Hepatocytoma cells HC-04 seeded 80,000/well and infected with 40,000 sporozoites/well with fresh *P*. *berghei* GFP sporozoites or left uninfected. After 3 hrs HC-04 were stained with DRAQ5, harvested and analyzed by flow cytometer. (A) Uninfected and no stain HC-04 cells. (B) Uninfected and DRAQ5 stained cells. (C) Sporozoites infected and DRAQ5 stained cells. Stained cells were gated in FL-1A (GFP, 488 nm) and in FL-4A (DRAQ5, 640 nm).

**Fig 3:**
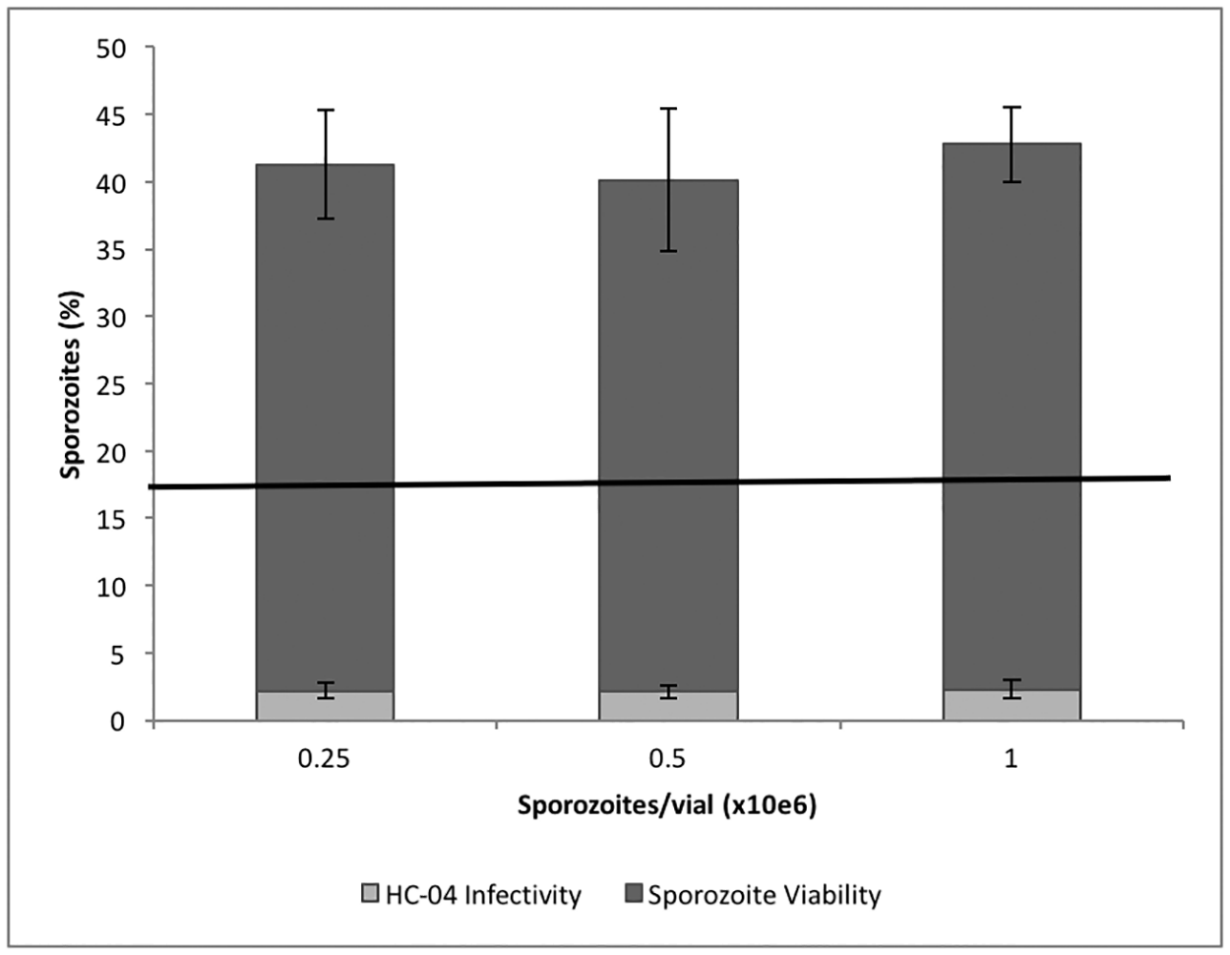
Capacity of *P*. *berghei* GFP sporozoites per vial cryopreserved in CryoStor CS2 and post-thaw infection of HC-04 cells. Sporozoites from same lot mixed with CryoStor CS2 (1:3 ratio), aliquoted as 100 μl/vial with varying concentrations (0.25, 0.5, 1 million), cryopreserved, thawed, diluted to adjust same numbers and infected to HC-04 cells at 40,000 sporozoites/well. A sample of fresh sporozoites used to infect HC-04 prior to cryopreservation in medium alone served as control (solid line) to calculate the relative sporozoite infectivity, used to estimate viability.

A GFP-expressing line of the rodent malaria parasite *P*. *berghei* was used for primary evaluation of protocols, using various solutions and conditions, to improve cryopreservation of infective salivary gland sporozoites. In this study, *P*. *berghei* GFP-expressing sporozoites were produced from infected mice fed to laboratory-reared *An*. *stephensi*. Sporozoites were isolated aseptically by salivary gland dissection and infectivity evaluated by *in vitro* assays before and after cryopreservation. The optimal standard conditions for evaluation of *in vitro* infectivity of sporozoites in HC-04 cells was 40,000 sporozoites/well for 3 hrs (Fig 1). A fast and efficient flow cytometer-based protocol was used to count parasite-infected HC-04 [15]. At the end of 3 hr incubation with sporozoites, HC-04 cultures were stained with DRAQ5 nuclear stain, harvested by trypsin treatment, and counted by flow cytometry. Infected HC-04 gated as a clearly separate population from uninfected HC-04 (Fig 2). Additional experiments, using only CryoStor CS2, were performed to assess the impact of sporozoite number per vial on cryopreservation efficiency and infection. In a fixed volume of 100μl per cryogenic storage vial, three concentrations of sporozoites (0.25/0.5/1.0 x 10^6^/100μl) were analyzed and results showed no significant difference (Fig 3). Therefore, the lowest concentration of 0.25 x 10^6^ sporozoites/vial was used, since this was considered sufficient for subsequent sporozoite evaluation assays.

### Evaluation of commercial cryopreservative solutions

**Fig 4:**
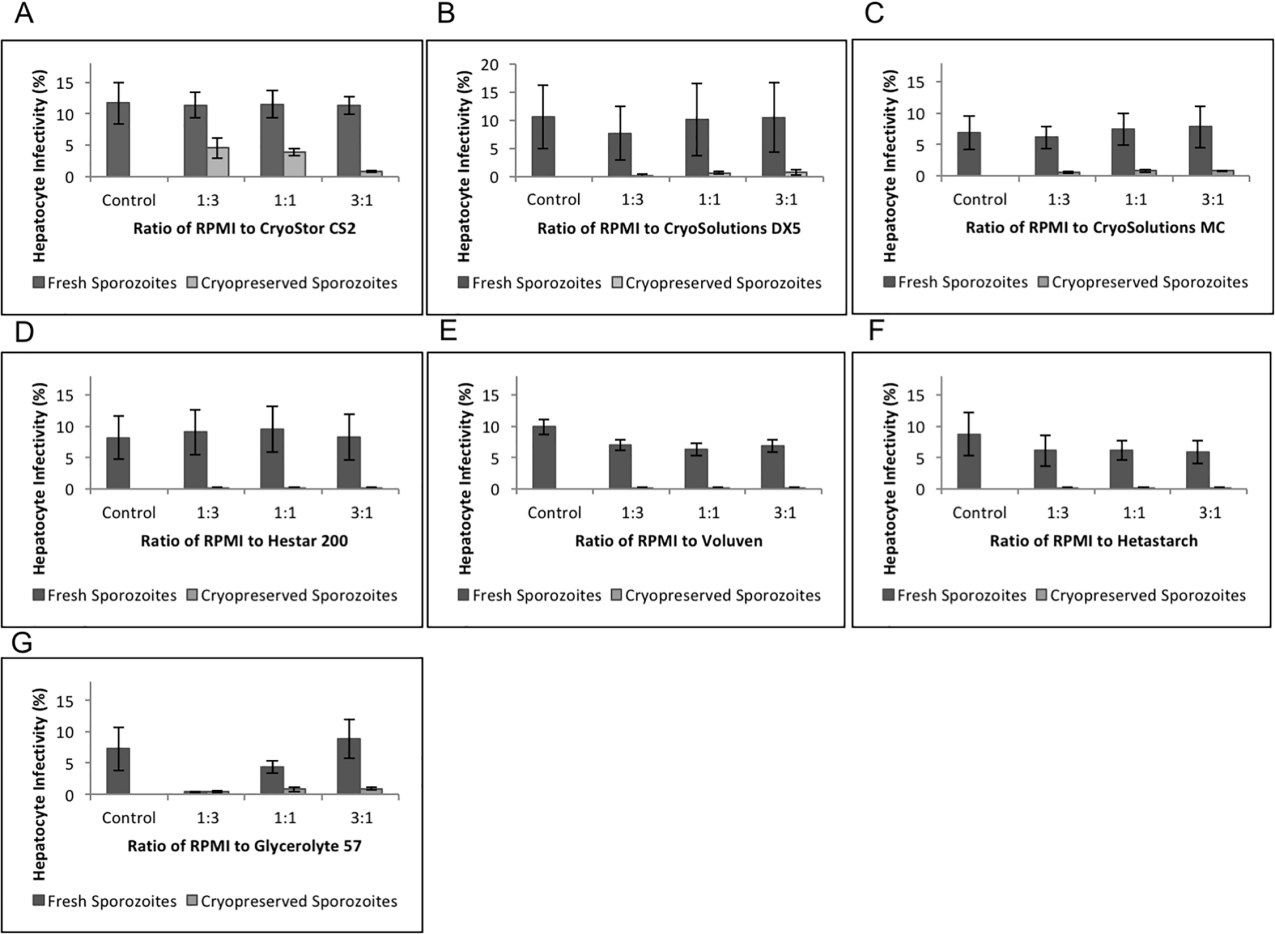
Cryopreservation of *P*. *berghei* GFP sporozoites in different cryoprotectants and evaluation of their infectivity for HC-04 cells. HC-04 cell infectivity of fresh sporozoites (40,000 sporozoites/well) was evaluated either in medium alone (control) or when mixed with cryoprotectants at different ratios (1:3, 1:1, 3:1) (dark bars). Subsequently, sporozoites from the same lot were cryopreserved in different cryoprotectants at same ratios and were used post-thaw to infect HC-04 cells (40,000 sporozoites/well) (light bars). In both cases samples were further diluted with medium to adjust given numbers. Cells were incubated for 3 hrs, harvested and analyzed by flow cytometer. Relative sporozoite infectivity was calculated as percent infected HC-04 versus control. (A) CryoStor CS2, (B) CryoSolutions DX5, (C) CyoSolutions MC, (D) Hestar 200, (E) Voluven, (F) Hetastarch, (G) Glycerolyte 57.

Seven commercially available cryopreservative solutions were selected based on their use in cryopreserving a diversity of cell types and tissues. Each solution contains at least one active component (DMSO, hydroxyethyl starch or glycerol) to serve as a cryoprotective agent when freezing cells and tissues (Table 1). The effectiveness of each cryopreservative solution was evaluated at three different ratios (1:3, 1:1, 3:1) to determine best possible combination (Fig 4). Simultaneously, the potential toxic effect of each cryopreservative solution was also evaluated by incubation of fresh sporozoites. In all cases, fresh and thawed cryopreserved sporozoites were used to infect HC-04 to determine sporozoite infectivity compared to aliquots of fresh sporozoites from the same batch in RPMI without any cryopreservative solution. The 1:3 ratio of sporozoites to CryoStor CS2 provided the best overall post-thaw recovery, which was significantly higher than any other cryopreservative solution evaluated. An independent replicate of all cryopreservative solutions at the 1:3 ratio confirmed the superiority of the CryoStor CS2 for cryopreserving *Plasmodium* sporozoites (Table 2).

**Table 2:** Summary of cryogenic solutions, post-thaw recovery and viability of *P*. *berghei* GFP sporozoites based on HC-04 cells infection.

Cryogenic Solutions	Thaw Method	% Sporozoite Recovery (Average±SD)^b^	% HC-04 Cell Infectivity (Average±SD)^c^	p-values^e^	% Relative Sporozoite Infectivity (Average±SD)^d^	p-values^e^
CryoStor CS2	Control	-	5.1 ±0.9	0.17	-	0.02
	RT^a^	97.6 ±3.4	2.0 ±0.5		37.9 ±5.6	
	37C	98.7 ±2.7	1.7 ±0.2		31.5 ±3.8	
CryoSolutions DX5	Control	-	7.4 ±0.4	0.21	-	0.21
	RT	67.0 ±5.0	0.1 ±0.04		1.7 ±0.5	
	37C	58.7 ±3.2	0.1 ±0.05		1.4 ±0.7	
CryoSolutions MC	Control	-	7.5 ±3.1	0.17	-	0.005
	RT	89.5 ±11.7	0.4 ±0.1		6.2 ±1.2	
	37C	85.7 ±10.8	0.3 ±0.1		4.4 ±0.6	
Hestar 200	Control	-	5.6 ±0.2	0.009	-	0.009
	RT	93.2 ±0.8	0.3 ±0.1		4.9 ±1.8	
	37C	97.5 ±0.4	0.5 ±0.1		8.4 ±2.4	
Voluven	Control	-	7.1 ±0.9	0.17	-	0.12
	RT	84.3 ±0.5	0.04 ±0.03		0.6 ±0.4	
	37C	81.8 ±14.0	0.06 ±0.02		0.8 ±0.2	
Hetastarch	Control	-	7.1 ±1.7	0.09	-	0.002
	RT	81.5 ±12.1	0.5 ±0.2		6.9 ±1.2	
	37C	89.9 ±14.2	0.9 ±0.4		12.3 ±3.4	
Glycerolyte 57	Control	-	5.1 ±0.5	0.21	-	0.21
	RT	84.2 ±4.7	0.3 ±0.1		6.6 ±2.3	
	37C	80.0 ±9.1	0.2 ±0.08		4.9 ±1.7	

^a^RT = Room temperature

^b^Percent sporozoites recovery determined by counting recovered sporozoites post-thaw as compared to prior cryopreservation.

^c^ Percent hepatocyte infectivity based on number of sporozoite infected HC-04 relative to the total counted in the respective well after 3 hr.

^d^Relative sporozoite infectivity calculated as percent HC-04 infected by cryopreserved sporozoites relative to fresh sporozoites.

^e^p-values calculated by one-way ANOVA for normally distributed data and by Kruskal-Wallis for non-normally distributed data. Anderson-Darling was used to determine the data distribution for each cryogenic solution.

**Fig 5:**
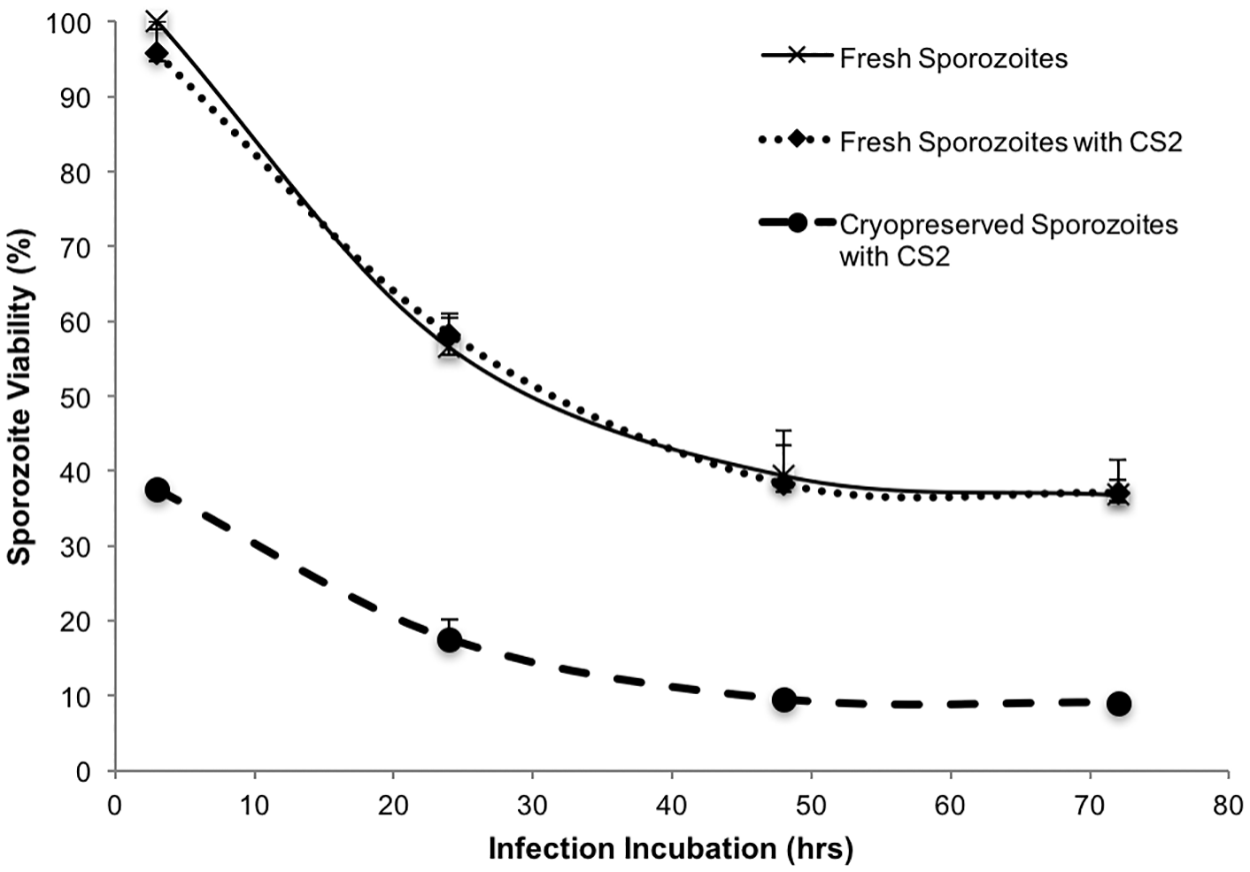
Infection, growth and development of fresh and CryoStor CS2 cryopreserved *P*. *berghei* GFP sporozoites in HC-04 cells. Fresh sporozoites from the same lot were used to infect HC-04 cells either in medium alone, mixed with CryoStor CS2 (1:3), or mixed and cryopreserved in CryoStor CS2 (1:3). In each group, sporozoites were diluted with medium to adjust given numbers (40,000 sporozoites/well). Sporozoites were incubated for 3 hrs, washed to remove un-invaded sporozoites and incubated further for 3 days. At each time point infected cells harvested and analyzed by flow cytometry. For this experiment sporozoite viability was calculated with the number of HC-04 infected with fresh sporozoites without CryoStor CS2 for 3 hrs used was the control for all time points fresh and cryopreserved. Fresh sporozoites only (solid line), fresh sporozoites with CryoStor CS2 (dotted line), cryopreserved sporozoites with CryoStor CS2 (dashed line).

In the extended experiments when additional ratios (3:1) were evaluated, CryoStor CS2, which had maximum infectivity at 1:3 ratio, hepatocyte infectivity at the 1:1 ratio was slightly lower while the 3:1 ratio was lowest (Fig 4A), suggesting a lack of protective efficacy from DMSO. In the same experiment, other results indicated CryoStor CS2 was not toxic to fresh sporozoites, since there was no significant difference in infectivity of fresh sporozoites in medium with or without CryoStor CS2 (Fig 5). However, the higher concentrations of DMSO, DX5 (5%) and MC (10%), clearly showed a slight toxic effect of DMSO at higher ratio concentrations post-thaw. There was lower infectivity of thawed cryopreserved sporozoites at each ratio compared to CryoStor CS2, including evident increased toxicity at higher concentration for fresh sporozoites for both DX5 and MC (Fig 4B and 4C). The solutions containing 6% hydroxyethyl starch (Hestar 200, Voluven, Hetastarch) retained good recovery of sporozoite numbers after thawing but resulted in very low preservation of sporozoites infectivity post-thaw. In addition, the Voluven and Hetastarch solutions exhibited moderate toxicity while Hestar 200 appeared to have low toxicity when mixed with fresh sporozoites (Fig 4D, 4E and 4F, respectively). Glycerolyte 57, the only glycerol cryopreservative solution evaluated, containing 50–60% glycerol, exhibited the highest toxicity effect when mixed with fresh sporozoites at ratios 1:3 and 1:1. However, Glycerolyte 57 had no measurable toxic effect at 3:1 ratio, but also had little protective efficacy in cryopreserving sporozoites (Fig 4G).

### HC-04 infectivity of cryopreserved sporozoites in *in vitro* assays

The *in vitro* infectivity results clearly indicated that CryoStor CS2 at ratio of 1:3 was the best solution evaluated to maintain post-thaw infectivity of cryopreserved sporozoites. Therefore, additional experiments were performed with this CryoStor CS2 protocol to evaluate the phenotypic effect on sporozoite growth and development for the *in vitro* and *in vivo* hepatic stages of this rodent malaria parasite. Similar to previous experiments, fresh sporozoites from the same lot were used to infect HC-04 either in medium alone, mixed with CryoStor CS2, or cryopreserved with CryoStor CS2 and then analyzed post-thaw. These analyses were done at similar dilution that was achieved after post-thaw with cryopreserved sporozoites and mixed with CS2 in multiple wells in each group. Infected cells were harvested in 4 replicates in each group at each time point (3 hrs, day 1, day 2, day 3). For this experiment, sporozoite viability, referred to as relative sporozoite infectivity, was calculated as the number of infected HC-04 cells, using number of HC-04 infected with fresh sporozoites without CryoStor CS2 as the control for all time points. This made it possible to calculate the effect of CryoStor CS2 on fresh and cryopreserved sporozoites. We defined 100% viability as the viability equal to that of fresh sporozoites without CyoStor CS2.

**Fig 6:**
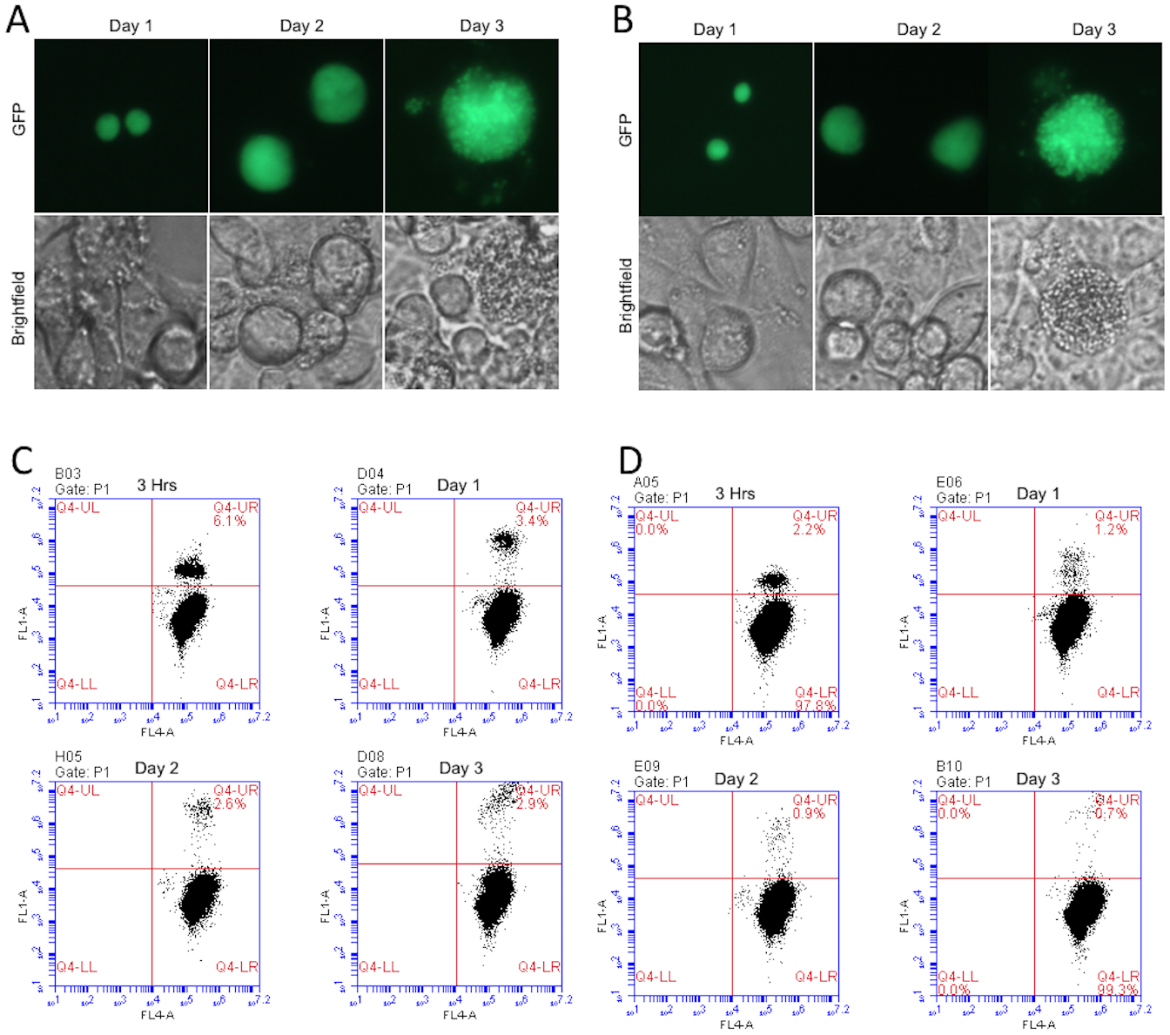
Infection, growth and development of *P*. *berghei* GFP sporozoites in HC-04 cells. Live images and IFA images of (A) fresh and (B) CryoStor CS2 cryopreserved *P*. *berghei* GFP sporozoites from day 1, day 2, day 3. Flow cytometer reading data from (C) fresh and (D) CryoStor CS2 cryopreserved *P*. *berghei* GFP sporozoites at 3 hrs, day 1, day 2, day 3. Stained cells were gated in FL-1A (GFP, 488 nm) and in FL-4A (DRAQ5, 640 nm).

The course of infection of fresh sporozoites in medium with and without CryoStor CS2 for invasion, growth and development of *in vitro* liver stage parasites was nearly identical (Fig 5). This statistical similarity in growth and development further supported the lack of toxicity of CryoStor CS2 on *P*. *berghei* sporozoite infectivity. Although the infectivity of cryopreserved sporozoites was significantly lower (40%), the progression rate for growth and development appeared similar in both fresh sporozoite groups (Fig 5). There was no significant difference in the progression rate for growth and development of cryopreserved sporozoites that did infect HC-04 hepatocytes (Table 3). All 3 groups of parasites produced viable infection, growth and development of mature stages at the same rate and time as indicated in (Fig 6). Mature schizonts in both fresh and CS2 cryopreserved sporozoites groups ruptured to release merosomes on day 3.

**Table 3:** Progression rate of fresh and CryoStor CS2 cryopreserved *P*. *berghei* GFP sporozoites invasion, growth and development in HC-04 cells.

Infection Incubation Period	Progression Rate (%)			Method of Calculation
	Fresh Sporozoites^a^	Fresh Sporozoites with CryoStor CS2	Cryopreserved Sporozoites with CryoStor CS2	
3 hrs	Control	Control	Control	
Day 1	56.49	60.84	46.59	Day1/3 hrsx100
Day 2	69.77	65.67	53.90	Day2/Day1x100
Day 3	93.45	96.87	95.73	Day3/Day2x100

^a^Progression rate was calculated from average sporozoite-infected HC-04 cells.

### HC-04 infectivity of cryopreserved sporozoites in *in vivo* assays

To analyze *in vivo* infectivity, laboratory mice were injected with fresh sporozoites diluted in medium alone or with cryopreserved sporozoites mixed with CryoStor CS2. The mice receiving each type of sporozoite were separated into three groups based on the single dose inoculum of 10,000, 20,000 or 40,000 sporozoites. The results from two independent experiments indicated cryopreserved sporozoites were infective in all groups (Table 4). The results indicated no significant differences in prepatent period and all mice (100%) in both fresh and cryopreserved groups became positive between day 4 and 5. Nonetheless, the *in vivo* infectivity of cryopreserved sporozoites may be marginally less than fresh sporozoites at the lower inoculum doses as there were slightly delayed prepatent periods for the 10,000 and 20,000 sporozoite doses. These blood stage infections in mice from cryopreserved sporozoites retained infectivity for mosquitoes and accordingly the next cycle of sporozoites development was comparable to fresh sporozoite infections.

**Table 4:** Infectivity of fresh and CryoStor CS2 cryopreserved *P*. *berghei* GFP sporozoites in mice.

Sporozoites/Mouse	Fresh Sporozoites		Cryopreserved Sporozoites^a^	
	No. of Mice Infected/Positive	Prepatent Period (days) (Average±SD)^b^	No. of Mice Infected/Positive	Prepatent Period (days) (Average±SD)^b^
10,000	4/4	4.50±0.58	4/4	5.00±0.00
20,000	4/4	4.50±0.58	4/4	4.75±0.50
40,000	4/4	4.00±0.00	4/4	4.00±0.00

^a^Sporozoites cryopreserved in CryoStor CS2 and thawed at room temperature.

^b^Prepatent period calculated from two experiments.

## Discussion

An important limitation for research on the sporozoite and liver stages of malaria parasites is access to infective sporozoites, especially for the human malaria parasites. These limitations can be due to lack of suitable laboratory infrastructure as well as the resource-intensive nature of maintaining the ongoing infection cycles of parasite and mosquitoes. Efficient cryopreservation of sporozoites could help increase access amongst laboratories by sharing a common source plus potentially more efficient management of laboratory experiments. To be effective, a cryopreservation method for *Plasmodium* sporozoites should retain adequate sporozoite infectivity, characteristic completion of liver stage development and production of infective blood-stage parasites. In this study, we have moved closer to this goal by validation of a cryopreservation protocol for *P*. *berghei* sporozoites that retains *in vitro* and *in vivo* sporozoite infectivity at approximately 40% of fresh sporozoites and sporozoites that do infect hepatocytes and complete liver stage development at the same rate as fresh sporozoites. A 2.5-fold decrease in infectivity appears to be in a similar range as for studies with cryopreserved *P*. *falciparum* sporozoites [16, 17]. In the current study, the commonly used practices employed to standardize the freezing and thawing processes for this protocol can likely be improved upon to increase viability and infectivity without substantial changes in other parameters of the protocol. For this study, all cryopreserved sporozoites were maintained in cryovials within the liquid nitrogen vapor phase for no more than 36 days. There were no experiments to test the loss of viability over time in long-term storage as that was not our focus. By using more precise cooling procedures, especially a controlled rate freezer to minimize ice crystal formation during the critical point freezing temperatures, we expect that significant improvements in retaining viability and infectivity can be achieved. At that point, we may be able to address any future concerns with loss of viability for long-term storage of no more than 36 days.

CryoStor CS2 was the best solution identified of the seven different commercially-available cryopreservation solutions we evaluated and had the lowest concentrations of DMSO of the three cryoprotective solutions evaluated. The optimal ratio of sporozoites in medium to CryoStor CS2 solution was 1:3 resulted in a final concentration of DMSO of 1.5%. Hepatocyte infectivity of fresh sporozoites incubated in this concentration of CryoStor CS2 solution was not significantly different than sporozoites incubated in the same medium without CryoStor CS2 when infectivity was tested *in vitro* in HC-04 cells or *in vivo* in mice, indicating that there was no DMSO toxicity at this concentration. However, toxicity was evident with similar analyses using the other DMSO solutions, DX5 (with 5% DMSO) and MC (with 10% DMSO), especially at higher ratio concentrations. The other cryoprotective solutions yielded poor outcomes at all concentrations tested, similar to earlier studies of *P*. *vivax* and *P*. *falciparum* [10].

The experimental model used in this study showed reproducible results. The results demonstrate successful cryopreservation of *P*. *berghei* sporozoites in CryoStor CS2 with excellent recovery and high infectivity in HC-04 hepatocytes *in vitro* and further validated in mice. The protocol developed is very simple using commercially-available, serum free cryopreservative solutions without using any expensive equipment or complicated technical steps that can be reproduced at any lab in the world. This simple method can hopefully expand availability of sporozoites and opportunities to perform research directed against sporozoites and liver stage model for chemotherapeutic and vaccine intervention studies prior to human trials. Future efforts will be directed towards using automated equipment to optimize cooling rate with various combination of cryopreservative solutions with additives to enhance infectivity of cryopreserved sporozoites.

## Supporting information

S1 Appendix Pb sporozoite cryo raw dataset. The Excel file with the raw data collected and the initial data analysis.
(XLSX)

## Abbreviations

DMSO: Dimethyl sulfoxide
GFP: Green fluorescence protein
